# Genome-Wide Identification and Expression Analysis of the Cucumber FKBP Gene Family in Response to Abiotic and Biotic Stresses

**DOI:** 10.3390/genes14112006

**Published:** 2023-10-27

**Authors:** Dekun Yang, Yahui Li, Mengdi Zhu, Rongjing Cui, Jiong Gao, Yingjie Shu, Xiaomin Lu, Huijun Zhang, Kaijing Zhang

**Affiliations:** 1 College of Agriculture, Anhui Science and Technology University, Fengyang 233100, China; dekun_yang1@163.com (D.Y.); 19505500698@163.com (M.Z.); 13969992371@163.com (R.C.); jionggao6@gmail.com (J.G.); shuyj@ahstu.edu.cn (Y.S.); luxm@ahstu.edu.cn (X.L.); 2 School of Life Science, Huaibei Normal University, Huaibei 235000, China; liyahui5651587@163.com

**Keywords:** cucumber, FKBP, gene family, expression analysis, abiotic and biotic stresses

## Abstract

The FKBP (FK506-binding protein) gene family is an important member of the PPlase protease family and plays a vital role during the processes of plant growth and development. However, no studies of the FKBP gene family have been reported in cucumber. In this study, 19 FKBP genes were identified in cucumber, which were located on chromosomes 1, 3, 4, 6, and 7. Phylogenetic analysis divided the cucumber FKBP genes into three subgroups. The FKBP genes in the same subgroup exhibited similar structures and conserved motifs. The *cis*-acting elements analysis revealed that the promoters of cucumber FKBP genes contained hormone-, stress-, and development-related *cis*-acting elements. Synteny analysis of the FKBP genes among cucumber, *Arabidopsis*, and rice showed that 12 kinds of syntenic relationships were detected between cucumber and *Arabidopsis* FKBP genes, and 3 kinds of syntenic relationships were observed between cucumber and rice FKBP genes. The tissue-specific expression analysis showed that some FKBP genes were expressed in all tissues, while others were only highly expressed in part of the 10 types of tissues. The expression profile analysis of cucumber FKBP genes under 13 types of stresses showed that the *CsaV3_1G007080* gene was differentially expressed under abiotic stresses (high temperature, NaCl, silicon, and photoperiod) and biotic stresses (downy mildew, green mottle mosaic virus, *Fusarium* wilt, *phytophthora capsica*, angular leaf spot, and root-knot nematode), which indicated that the *CsaV3_1G007080* gene plays an important role in the growth and development of cucumber. The interaction protein analysis showed that most of the proteins in the FKBP gene family interacted with each other. The results of this study will lay the foundation for further research on the molecular biological functions of the cucumber FKBP gene family.

## 1. Introduction

Multiple proteins, such as transcription factors, protein kinases, and immunophilins, have been reported to be involved in the responses to various stresses in plants [1,2,3]. Immunophilin is a cellular receptor protein of immunosuppressive drugs, which binds FK506, cyclosporin-A (CsA), and rapamycin [4]. According to the sensitivity to immunosuppressive drugs, immunophilin has been classified into two subfamilies as cyclophilin (cyclosporin A-binding protein) and FKBP (FK506/rapamycin-binding protein). FKBP is not sensitive to any of these immunosuppressive agents [5]. FKBP belongs to the peptidyl-proline *cis*-*trans* isomerase (PPlase) superfamily [6] and has been implicated in a wide spectrum of biological processes, including protein folding, hormone signaling, growth and development, and stress responses, which are widely found in bacteria, fungi, plants, and animals [7,8,9,10]. Every FKBP gene contains at least one FK506-binding domain (FKBd), a conserved peptide sequence of about 110 amino acids also known as FKBP12 [11,12]. Single-domain (low molecular weight) FKBPs have a single FKBd, while multidomain (high molecular weight) FKBPs contain up to three FKBds, along with tetratricopeptide repeat (TPR), coiled-coil domain (CCD), or C-terminal calmodulin-binding domains (CaM-BDs) for protein–protein interactions or recognition or for the assembly of multiprotein complexes [13,14].

The FKBP gene family has been reported in a lot of plant species with the further development of plant genome sequencing technology [15]. For example, 22, 29, 30, 24, 71, 23, 21, and 38 FKBP genes have been identified in *Arabidopsis* [4], rice [16], maize [17], tomato [18], wheat [19], strawberry [20], peach [21], and apple [22], respectively. The FKBP genes regulate stress responses [23,24], growth and development [25], photosynthesis [26], and the expression levels of other genes [27]. In *Arabidopsis*, *ROF1* (*AtFKBP62*) and *ROF2* (*AtFKBP65*) responded to high-temperature stress and affected the accumulation of the heat shock transcription factor HsfA2. The overexpression of maize FKBP gene *ZmFKBP20-1* significantly enhanced the tolerances to drought and salt in *Arabidopsis* [17]. The functional destruction of the *AtFKBP42* gene caused a dwarf phenotype with additional disorientated growth of all organs [28]. In wheat, transgenic lines overexpressing *wFKBP77* showed major morphological abnormalities, specifically relating to height, leaf shape, spike morphology, and sterility, and the grain weight and composition were altered after overexpressing the *wFKBP73* gene [29]. In rice, the *OsFKBP20-1a* gene was significantly upregulated after desiccation treatments, while the expression level of the *OsFKBP20-1b* gene was increased under salt and desiccation stresses [30]. The *AtFKBP65* gene could induce callose accumulation in the plant cell wall for preventing the infection of *Pseudomonas syringae* [23]. Taken together, the FKBP gene family plays different roles in plant growth and development and the response to stress.

Cucumbers (*Cucumis sativus* L.) are one of the most important vegetable crops worldwide [31,32]. The cucumber genome data became publicly available as early as 2009 [33]. Along with the rapid development of next-generation sequencing technology, the cucumber genome is constantly being updated and has been updated now to ChineseLong_V3 [34]. Many gene family studies have been carried out using the published high-quality genomic information of cucumber, such as WRKY [35], Histone [36], B-BOX [37], CLE [38], and so on. However, studies on the cucumber FKBP gene family have not been performed. In this study, 19 FKBP genes in cucumber were identified through whole-genome identification, and then, the chromosomal location, gene structure, phylogeny, and synteny analyses were performed. To investigate the expression profiles of cucumber FKBP genes in different tissues and under different stresses, the publicly available cucumber transcriptome sequencing big data of different tissues and stresses were reanalyzed by combining with the newly published cucumber genome (ChineseLong_V3 version). This study will be helpful for understanding the biological functions of FKBP genes during cucumber growth and development and provide valuable information for the further functional verification of FKBP genes and molecular breeding in cucumber.

## 2. Materials and Methods

### 2.1. Identification and Chromosomal Distribution of Cucumber FKBP Genes

The HMM file (PF00254) of the FKBP gene family was downloaded from the InterPro database [39], and the cucumber FKBP genes were retrieved from the cucumber genome database with the E value of 1 × 10^−5^ in HMMER [40]. The protein sequences of the corresponding FKBP genes were extracted using perl script. Subsequently, all the putative FKBP protein sequences were validated with the SMART [41] and NCBI [42] databases. The protein sequences of the cucumber FKBP family members were uploaded to the online website ExPASy [43] for the physicochemical characteristics analysis. The chromosomal location of each validated FKBP gene was extracted from the ChineseLong_V3 GFF3 file and mapped on the cucumber chromosomes using TBtools software (version 1.120) [44].

### 2.2. Phylogenetic Analysis of FKBP Family Genes from Cucumber, Arabidopsis, and Rice

The sequences of 19 cucumber FKBP proteins, 22 *Arabidopsis thaliana* FKBP proteins, and 29 rice FKBP proteins were retrieved and the phylogenetic analysis was performed using MEGA11 software (version 11.0.13) [45]. The maximum likelihood method was adopted to construct the phylogenetic tree with default parameters.

### 2.3. Gene Structure, Conserved Motif, and Cis-Acting Elements Analyses of Cucumber FKBP Genes

Using TBtools software, the structures of the cucumber FKBP genes were analyzed. The online website MEME [46] was used to analyze the conserved motifs of the cucumber FKBP family genes. The parameters in MEME were set as the maximum motif number was 10, and the optimum width of motif was 6–100. Finally, the structure and conserved motifs of the cucumber FKBP genes were visualized using TBtools. The *cis*-acting elements of the promoters of the cucumber FKBP family genes were analyzed within the 2.0 kb upstream sequences from the transcription start sites of the cucumber FKBP family genes using the online website PlantCare [47].

### 2.4. Synteny Analysis of FKBP Family Genes from Cucumber, A. thaliana, and Rice

The tandem and segmental duplications in the cucumber FKBP gene family were analyzed using TBtools software [44]. The collinearity relationships of the FKBP family genes from cucumber, *Arabidopsis*, and rice were also analyzed and visualized with TBtools software.

### 2.5. Tissue-Specific Expression Analysis of the Cucumber FKBP Family Genes

The published transcriptome sequencing data of different cucumber tissues (PRJNA80169) [48] were combined with the cucumber ChineseLong_V3 genome data to conduct the RNA-seq reanalysis, and then, the expression heatmap of the cucumber FKBP genes in different cucumber tissues was illustrated with TBtools software.

### 2.6. Expression Patterns Analysis of the Cucumber FKBP Family Genes under Various Stresses

In total, 13 types of stresses, including 5 types of abiotic stresses (high temperature (PRJNA634519) [35], salt and silicon (PRJNA477930) [49], waterlogging (PRJNA678740) [50], photoperiod (PRJNA475903) [51], and different ratios of blue and red light (PRJNA476021) [52]) and 8 types of biotic stresses (downy mildew (PRJNA285071) [53], powdery mildew (PRJNA321023) [54], *Prunus* necrotic ringspot virus (PRJNA837466) [55], green mottle mosaic virus (PRJNA646644) [56], *Fusarium* wilt (PRJNA472169) [57], *Phytophthora capsici* (PRJNA345040) [58], angular leaf spot (PRJNA704621) [59], and root-knot nematode (PRJNA419665) [60]) were downloaded and used for the expression patterns analysis of cucumber FKBP family genes. The published cucumber transcriptome data under different stresses were combined with the cucumber V3 genome data for the RNA-seq reanalysis. Finally, expression heatmaps of the cucumber FKBP family genes in response to different stresses were drawn with TBtools software.

### 2.7. Protein Interaction Analysis of FKBP Family Gene in Cucumber

The ChineseLong_V3 protein sequences and the cucumber FKBP family protein sequences were uploaded to the online website STRING (http://string-db.org/cgi (accessed on 25 March 2023)), and then, the target protein *CsaV3_1G007080* and the cucumber FKBP proteins were analyzed for protein interaction prediction.

## 3. Results

### 3.1. Identification and Physicochemical Characteristics of Cucumber FKBP Genes

In this study, 19 FKBP family genes were identified in the cucumber genome (Chinese Long_V3). The CDS sizes ranged from 339 (*CsaV3_7G006970*) to 1866 (*CsaV3_4G025730*) bp, encoding a number of amino acids ranging from 112 (*CsaV3_7G006970*) to 621 (*CsaV3_4G025730*), and the molecular weight varied from 11.94 (*CsaV3_7G006970*) to 69.96 (*CsaV3_4G025730*) kD. The theoretical isoelectric points of the 19 FKBP proteins were between 5.23 (*CsaV3_3G015840*) and 9.49 (*CsaV3_4G037510*). The instability index analysis showed that eight FKBP proteins were unstable (instability coefficient < 40), including *CsaV3_3G015840*, *CsaV3_3G032060*, *CsaV3_4G002230*, *CsaV3_6G001250*, *CsaV3_6G053090*, *CsaV3_7G006970*, *CsaV3_7G026570*, and *CsaV3_7G026580*, and the remaining FKBP proteins were stable. The aliphatic indexes of the cucumber FKBP proteins were between 61.28 (*CsaV3_3G016330*) and 93.77 (*CsaV3_7G023830*). The grand average hydropathicity values of all the FKBP proteins were less than zero, indicating that all the FKBP proteins in the cucumber were hydrophilic. The prediction of subcellular localization revealed that nine FKBP genes were located in chloroplast, and the remaining FKBP genes were distributed in the nucleus, cytoplasm, cytoskeleton, and peroxisome, respectively (Table 1).

### 3.2. Chromosome Distribution of Cucumber FKBP Genes

**Figure 1:**
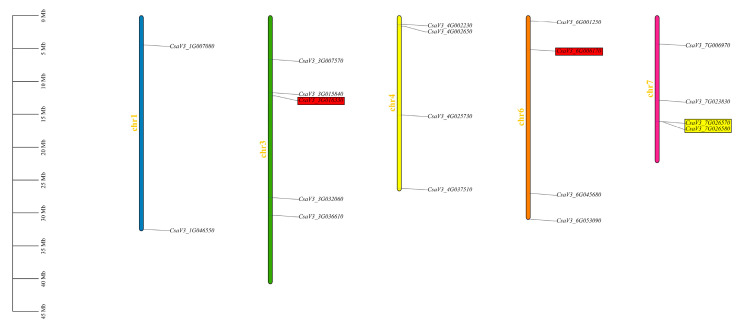
Distribution of the cucumber FKBP family genes on the chromosomes. The genes marked in yellow color were tandem duplication gene pairs, and the genes marked in red color were segmental duplication gene pairs.

Based on the chromosomal locations of the cucumber FKBP family genes, a distribution map of the cucumber FKBP genes on the chromosomes was drawn. The results showed that 19 FKBP genes were distributed on chromosomes 1, 3, 4, 6, and 7, respectively. The largest number of FKBP genes (five FKBP genes) were mapped on chromosome 3. Four FKBP genes were distributed on chromosomes 4, 6, and 7, respectively, and only two FKBP genes were distributed on chromosome 1. Among them, the *CsaV3_3G016330* gene on chromosome 3 and *CsaV3_6G006170* gene on chromosome 6 were segmental duplication gene pairs. The *CsaV3_7G026570* and *CsaV3_7G026580* genes on chromosome 7 were tandem duplication gene pairs (Figure 1).

### 3.3. Phylogenetic Analysis of FKBP Family Genes in Cucumber, Arabidopsis, and Rice

**Figure 2:**
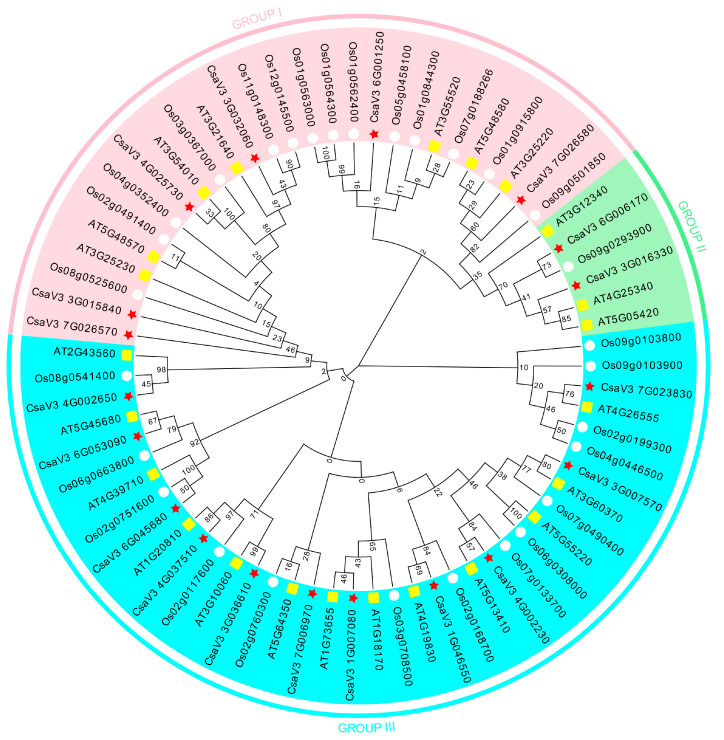
Phylogenetic analysis of the FKBP gene family from cucumber, *Arabidopsis*, and rice.

The phylogenetic tree was constructed with the FKBP proteins from cucumber, *Arabidopsis*, and rice (Figure 2). The results showed that the phylogenetic tree was divided into three subfamilies, such as GROUP I, II, and III. There were 27 FKBP genes in GROUP I, 6 FKBP genes in GROUP II, and 38 FKBP genes in GROUP III. The phylogenetic analysis revealed that nine pairs of orthologous FKBP genes were found between cucumber and *Arabidopsis*, including *CsaV3_4G025730*/*AT3G54010*, *CsaV3_7G023830*/*AT4G26555*, *CsaV3_3G007570*/*AT3G60370*, *CsaV3_4G002230*/*AT5G13410*, *CsaV3_1G046550*/*AT1G19930*, *CsaV3_1G007080*/*AT1G73655*, *CsaV3_3G036610*/*AT3G10060*, *CsaV3_4G037510*/*AT1G20810*, and *CsaV3_6G053090*/*AT5G45680*, and three pairs of orthologous FKBP genes were found between cucumber and rice, such as *CsaV3_6G006170*/*Os09g0293900*, *CsaV3_6G045680*/*Os02g0751600*, and *CsaV3_4G002650*/*Os08g0541400*. The FKBP genes with similar evolutionary relationships were similar in gene structure and function; thus, the biological functions of the cucumber FKBP genes could be predicted based on studies of the gene functions of the FKBP genes in *Arabidopsis* and rice.

### 3.4. Gene Structure and Conserved Motif Analysis of Cucumber FKBP Genes

**Figure 3:**
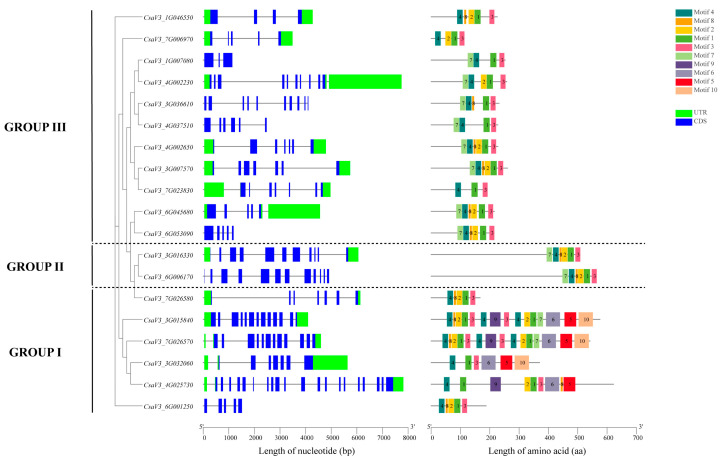
Schematic diagram of the exon–intron structures of FKBP genes and conserved motifs of FKBP proteins in cucumber.

According to the structural diagram of the cucumber FKBP family genes (Figure 3), there were 6 cucumber FKBP genes in GROUP I, 2 cucumber FKBP genes in GROUP II, and 11 cucumber FKBP genes in GROUP III. The average number of exons and introns in GROUP II were the highest at 11 and 10.5, respectively, while the average number of exons and introns in GROUP III were the lowest at 6.23 and 5.55, respectively. GROUP I contained an average of 10.67 exons and 10.12 introns. The online software MEME (version 5.1.0) was used to analyze the conserved motifs in the cucumber FKBP genes, and 10 motifs were obtained (Table 2). The conserved motif analysis showed that the FKBP genes in GROUPS II and III contained the same ordered motifs: 7, 4, 8, 2, 1, and 3, which predicted that the FKBP genes in these two subfamilies may share similar biological functions. The motifs in GROUP I were significantly different from those in the other two subfamilies and significantly different within their subfamily, which indicated that the FKBP genes in GROUP I may be contributed to functional diversification.

### 3.5. Synteny Analysis of FKBP Genes among Cucumber, Arabidopsis, and Rice

**Figure 4:**
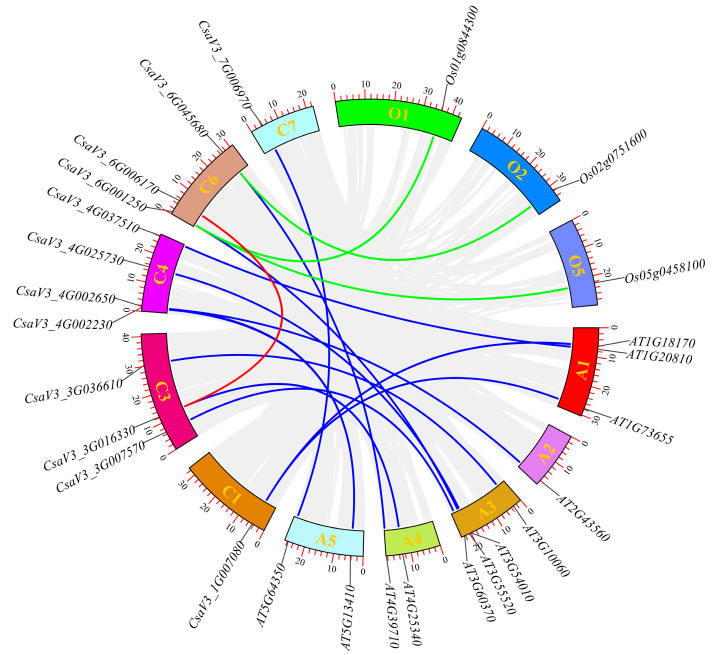
Syntenic relationships of the FKBP family genes among cucumber, *Arabidopsis*, and rice. The red lines represented the segmentally duplicated FKBP genes in cucumber. The blue lines represented the orthologous relationships of the FKBP genes between cucumber and *Arabidopsis*. The green lines represent the orthologous relationships of the FKBP genes between cucumber and rice.

Synteny analysis of the cucumber FKBP genes was performed to further understanding the evolution of the FKBP genes in cucumber (Figure 4). The results showed that there was only one segmental duplication (*CsaV3_3G036610*/*CsaV3_6G006170*) among the 19 FKBP genes in the cucumber. Twelve kinds of syntenic relationships were detected between 11 cucumber FKBP genes (*CsaV3_1G007080, CsaV3_4G037510, CsaV3_4G002650, CsaV3_3G036610, CsaV3_3G007570, CsaV3_4G025730, CsaV3_6G001250, CsaV3_3G016330, CsaV3_6G045680, CsaV3_4G002230,* and *CsaV3_7G006970*) and 12 *Arabidopsis* FKBP genes (*AT1G18170, AT1G73655, AT1G20810, AT2G43560, AT3G10060, AT3G60370, AT3G54010, AT3G55520, AT4G25340, AT4G39710, AT5G13410,* and *AT5G64350*). Three kinds of syntenic relationships were observed between two cucumber FKBP genes (*CsaV3_6G001250* and *CsaV3_6G045680*) and three rice FKBP genes (*Os01g0844300*, *Os02g0751600*, and *Os05g0458100*). The remaining seven cucumber FKBP genes (*CsaV3_1G046550*, *CsaV3_3G015840*, *CsaV3_3G032060*, *CsaV3_6G053090*, *CsaV3_7G023830*, *CsaV3_7G026570*, and *CsaV3_7G026580*) had no syntenic relationships with either *Arabidopsis* or rice.

### 3.6. Analysis of the Cis-Acting Elements in Cucumber FKBP Genes

**Figure 5:**
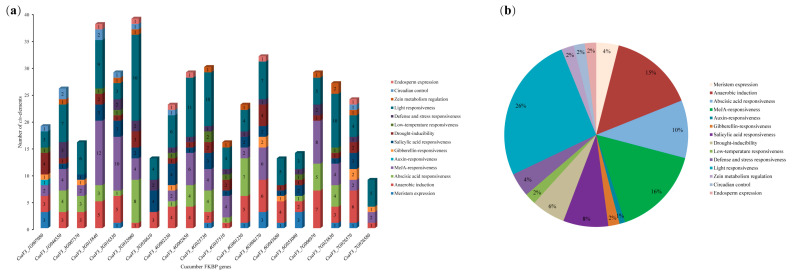
The *cis*-acting elements analysis of the promoters of cucumber FKBP genes. (**a**) The number of *cis*-acting elements in the promoter sequences of each cucumber FKBP gene. (**b**) The relative proportions of different *cis*-acting elements in the promoter sequence of cucumber FKBP genes.

A total of 14 types of *cis*-acting elements were identified in the promoter sequences of 19 cucumber FKBP genes (Figure 5). Among them, the light response and anaerobic response *cis*-acting elements were distributed in all 19 FKBP genes, and the auxin response *cis*-acting element was only distributed in the *CsaV3_1G007080* gene (Figure 5a). The hormone-related (auxin, abscisic acid, gibberellin, methyl jasmonate, and salicylic acid) *cis*-acting elements accounted for the highest proportion (37%), followed by the light responsiveness *cis*-acting elements (26%) (Figure 5b). In addition, *cis*-acting elements related to the stress (drought, low temperature, and defense) response, circadian control, endosperm expression, and meristem expression were also identified. These *cis*-acting elements might play corresponding roles during cucumber growth and development.

### 3.7. Tissue-Specific Expression Analysis of Cucumber FKBP Genes

**Figure 6:**
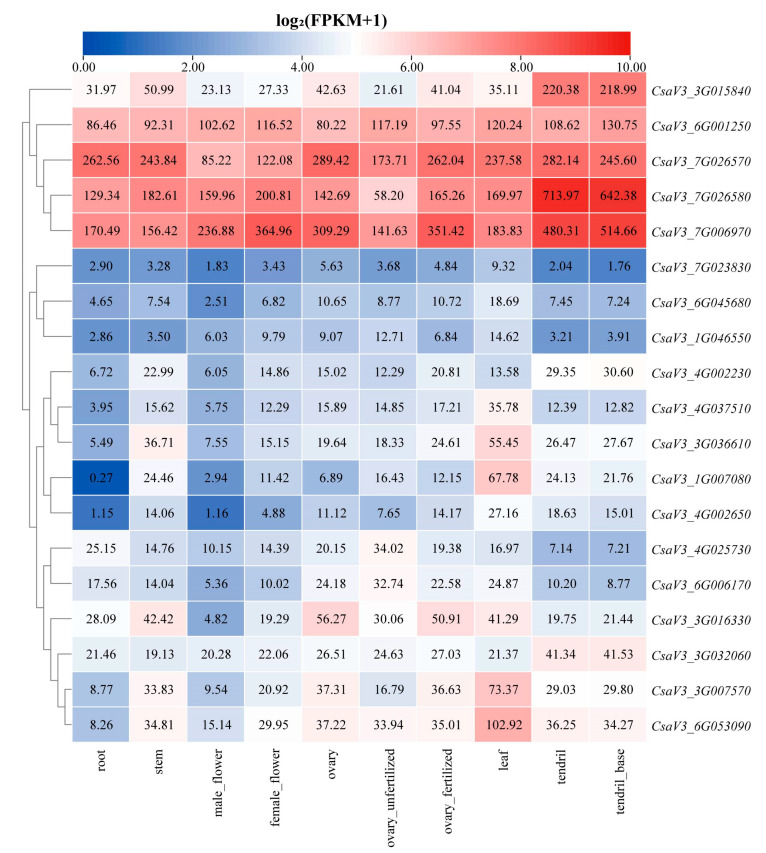
The expression profiling of cucumber FKBP genes in different tissues.

Tissue-specific expression analysis of the cucumber FKBP family genes showed that four FKBP genes, including *CsaV3_6G001250*, *CsaV3_7G026570*, *CsaV3_7G026580*, and *CsaV3_7G006970*, were highly expressed in 10 types of cucumber tissues, whereas the *CsaV3_7G023830*, *CsaV3_6G045680,* and *CsaV3_1G046550* genes were expressed at low levels in all tissues. Four FKBP genes, including *CsaV3_3G015840*, *CsaV3_7G026580*, *CsaV3_7G006970*, and *CsaV3_3G032060*, were specifically expressed in the tendrils and tendril base. Five FKBP genes, including *CsaV3_4G037510*, *CsaV3_3G036610*, *CsaV3_1G007080*, *CsaV3_3G007570*, and *CsaV3_6G053090*, were specifically expressed in the leaves. The *CsaV3_3G016330* gene was highly expressed in all tissues, with the exception of female flowers. In addition, the expression levels of the 19 FKBP genes in the female flowers were higher than those in the male flowers, which indicated that these FKBP genes may be involved in the formation of fruits (Figure 6).

### 3.8. Expression Patterns Analysis of Cucumber FKBP Genes under Abiotic Stresses

**Figure 7:**
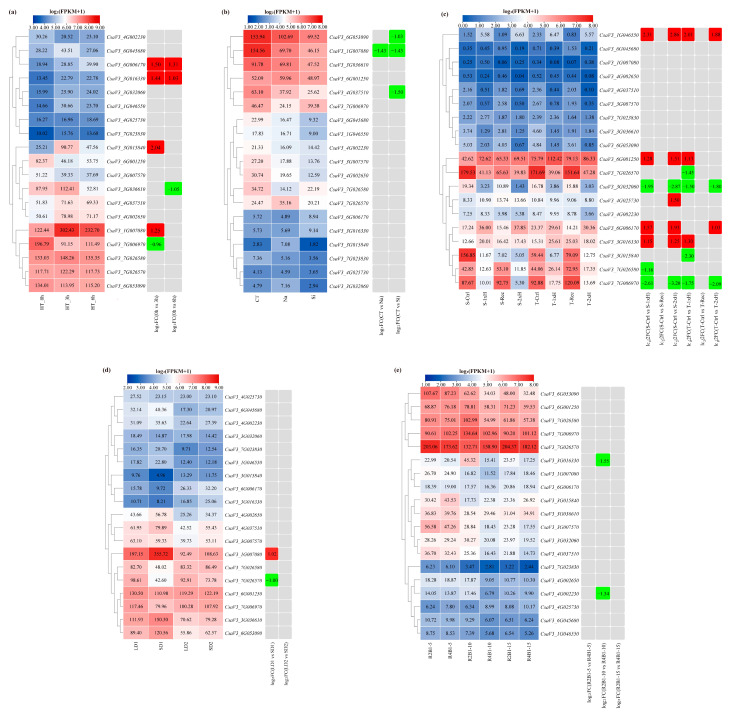
The expression patterns of cucumber FKBP genes in response to abiotic stresses. (**a**) The expression heatmap of cucumber FKBP genes in response to high-temperature stress. CT, HT_3h, and HT_6h: high-temperature treatment for 0, 3, and 6 h, respectively. (**b**) The expression heatmap of cucumber FKBP genes in response to salt and silicon stresses. CT: control treatment; Na: NaCl treatment; Si: silicon treatment. (**c**) The expression heatmap of cucumber FKBP genes in response to waterlogging stress. S: sensitive plant; R: resistant plant; Ctrl: untreated plants cultivated under optimal conditions; 1xH: non-primed plants waterlogged for 7 days only once; Rec: plants after 7 days of waterlogging and 14 days of recovery; 2xH: primed plants waterlogged for 7 days and after 14 days of recovery, then waterlogged again. (**d**) The expression heatmap of cucumber FKBP genes in response to photoperiod stress. LD1: long-day treatment for 7, 14, and 21 days; LD2: long-day treatment for 37 and 44 days; SD1: short-day treatment for 7, 14, and 21 days; SD2: short-day treatment for 37 and 44 days. (**e**) The expression heatmap of cucumber FKBP genes in response to different ratios of blue and red light stress. R2B1: red light: blue light = 2:1; R4B1: red light: blue light = 4:1; 5, 10, and 15: treatment for 5, 10, and 15 days, respectively. The data in the left expression heatmaps were the original FPKM values; the data in the right boxes were log_2_ (fold change) values highlighted by red (upregulation) and green (downregulation) colors.

To examine the expression patterns of cucumber FKBP genes under various abiotic stresses, the published cucumber transcriptome sequencing data of high temperature, salt and silicon, waterlogging, photoperiod, and different ratios of blue and red light were reanalyzed (Figure 7). Under high-temperature stress, the expression levels of the *CsaV3_7G006970* and *CsaV3_3G036610* genes were significantly reduced after 3 h and 6 h of high-temperature treatment, respectively. The expression levels of the *CsaV3_1G007080* and *CsaV3_3G015840* genes were only significantly increased after 3 h of high-temperature treatment, while the *CsaV3_3G016330* and *CsaV3_6G006170* genes were simultaneously significantly upregulated after 3 h and 6 h of high-temperature treatment (Figure 7a). Under the salt and silicon stresses, the expression level of the *CsaV3_1G007080* gene was significantly reduced under both salt and silicon stresses, while the expression levels of the *CsaV3_4G037510* and *CsaV3_6G053090* genes were only significantly reduced under silicon stress (Figure 7b). Under waterlogging stress, the expression levels of four FKBP genes, including *CsaV3_1G046550*, *CsaV3_6G001250*, *CsaV3_6G006170*, and *CsaV3_3G016330*, were significantly increased in both resistant and sensitive materials. The expression levels of the *CsaV3_3G032060* and *CsaV3_7G006790* genes were significantly reduced in both resistant and sensitive materials. The *CsaV3_4G025730* gene was only significantly upregulated in the sensitive material. The *CsaV3_7G026580* gene was only significantly downregulated in the sensitive material. The *CsaV3_7G026570* and *CsaV3_3G015840* genes were only significantly downregulated in the resistant material (Figure 7c). Under the photoperiod treatments, compared to the equal day treatment, only two differentially expressed FKBP genes were found after the short day photoperiod treatment. Compared to the control, the *CsaV3_1G007080* gene was significantly upregulated, and the *CsaV3_7G026570* gene was significantly downregulated (Figure 7d). Under the different ratios of blue and red light stress, significantly downregulated expression of the *CsaV3_3G016330* and *CsaV3_4G002230* genes occurred at 10 days after the red and blue light treatment (Figure 7e).

### 3.9. Expression Patterns Analysis of Cucumber FKBP Genes under Biotic Stresses

**Figure 8:**
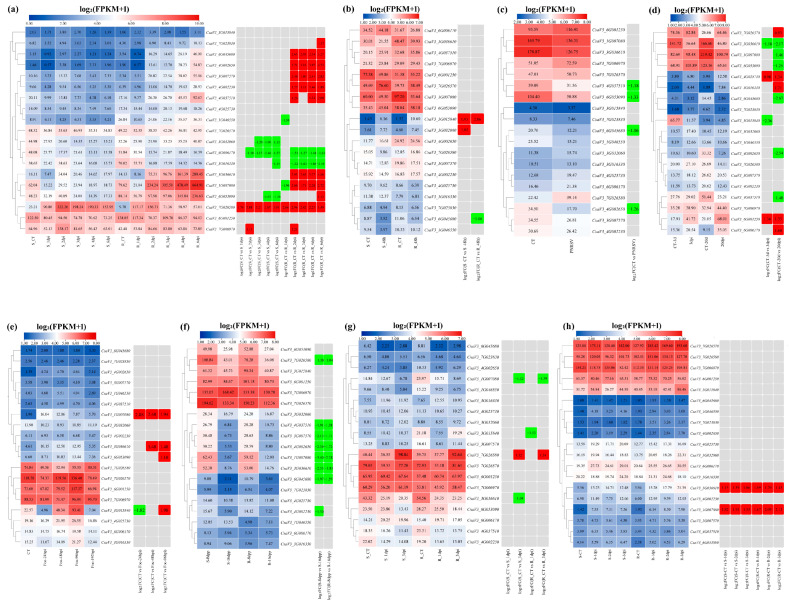
The expression patterns of cucumber FKBP genes in response to biotic stresses. (**a**) The expression heatmap of cucumber FKBP genes in response to downy mildew stress. S: susceptible plant; R: resistant plant; CT, 1 dpi, 2 dpi, 3 dpi, 4 dpi, and 6 dpi were 0, 1, 2, 3, 4, and 6 days post-inoculation, respectively. (**b**) The expression heatmap of cucumber FKBP genes in response to powdery mildew stress. S: susceptible plant; R: resistant plant; CT: control treatment; 48 h: 48 h post-inoculation. (**c**) The expression heatmap of cucumber FKBP genes in response to *Prunus* necrotic ringspot virus stress. CT: control treatment; PNRSV: inoculation with *Prunus* necrotic ringspot virus. (**d**) The expression heatmap of cucumber FKBP genes in response to cucumber green mottle mosaic virus stress. CT, 3 dpi, and 20 dpi: 0, 3, and 20 days post-inoculation, respectively. (**e**) The expression heatmap of cucumber FKBP genes in response to *Fusarium* wilt stress. FOC: *Fusarium* wilt treatment; CT, 2 hpi, 48 hpi, 96 hpi, and 192 hpi: 0, 2, 48, 96, and 192 h post-inoculation, respectively. (**f**) The expression heatmap of cucumber FKBP genes in response to *P. capsici* stress. S: susceptible plant; R: resistant plant; 8 dpp and 16 dpp: 8 and 16 days post-pollination, respectively. (**g**) The expression heatmap of cucumber FKBP genes in response to angular leaf spot stress. S: susceptible plant; R: resistant plant; CT, 1 dpi, and 3 dpi: 0, 1, and 3 days post-inoculation, respectively. (**h**) The expression heatmap of cucumber FKBP genes in response to root-knot nematode stress. S: susceptible plant; R: resistant plant; CT, 1 dpi, 2 dpi, and 3 dpi: 0, 1, 2, and 3 days post-inoculation, respectively. The data in the left expression heatmaps were the original FPKM values; the data in the right boxes were log_2_ (fold change) values highlighted by red (upregulation) and green (downregulation) colors.

To examine the expression patterns of cucumber FKBP genes under various biotic stresses, the available cucumber transcriptome sequencing data of downy mildew, powdery mildew, *Prunus* necrotic ringspot virus, green mottle mosaic virus, *Fusarium* wilt, *P. capsici*, angular leaf spot, and root-knot nematodes were reanalyzed by combining with the cucumber ChineseLong_V3 genome data, and the expression heatmaps were drawn using TBtools software (Figure 8). Under downy mildew stress, compared to the control, the expression level of the *CsaV3_3G032060* gene was significantly reduced in the susceptible material, the expression level of the *CsaV3_1G046550* gene was significantly reduced in the resistant material, and the expression level of the *CsaV3_6G053090* gene was significantly reduced in the susceptible material and significantly increased in the resistant material. Seven FKBP genes, including *CsaV3_7G023830*, *CsaV3_6G045680*, *CsaV3_4G002650*, *CsaV3_3G007570*, *CsaV3_4G002230*, *CsaV3_4G035710*, and *CsaV3_3G036610*, were significantly downregulated in the resistant material. The *CsaV3_7G026580* and *CsaV3_7G006970* genes were significantly upregulated in both resistant and susceptible plants, whereas the *CsaV3_6G006170* and *CsaV3_3G016330* genes were significantly downregulated in both resistant and susceptible plants. Notably, the *CsaV3_1G007080* gene was significantly downregulated after 1 day of treatment, followed by being upregulated in the resistant material (Figure 8a). Under powdery mildew stress, the *CsaV3_3G015840* gene was significantly upregulated in both resistant and susceptible materials, the *CsaV3_3G032060* gene was only significantly upregulated in the susceptible material, and the *CsaV3_6G045680* gene was only significantly downregulated in the resistant material (Figure 8b). Under infection of the *Prunus* necrotic ringspot virus, compared to the control, four FKBP genes, including *CsaV3_4G037510*, *CsaV3_6G053090*, *CsaV3_6G045680,* and *CsaV3_4G002650*, were significantly downregulated (Figure 8c). Under infection of the green mottle mosaic virus, compared to the control, two FKBP genes, including *CsaV3_4G025730* and *CsaV3_6G001250*, were significantly upregulated at 3 and 20 days post-infection, whereas the *CsaV3_3G036610* gene was significantly downregulated at 3 and 20 days post-infection. The *CsaV3_3G015840* gene was significantly downregulated at 3 days post-infection, while the *CsaV3_7G026570*, *CsaV3_3G016330*, and *CsaV3_6G006170* genes were significantly upregulated at 20 days post-infection. Five FKBP genes, including *CsaV3_1G007080*, *CsaV3_6G053090*, *CsaV3_6G045680*, *CsaV3_4G002650*, and *CsaV3_4G037510*, were significantly downregulated at 20 days post-infection (Figure 8d). Under *Fusarium* wilt stress, compared to the control, the *CsaV3_1G007080* gene was significantly upregulated at 24 hpi, 48 hpi, and 96 hpi (hours post-inoculation). The *CsaV3_3G036610* gene was significantly upregulated at 48 hpi and 96 hpi, while the *CsaV3_6G053090* gene was only significantly upregulated at 96 hpi. The *CsaV3_3G015840* gene was significantly downregulated at 24 hpi but significantly upregulated at 96 hpi (Figure 8e). Under infection of *P. capsici*, seven FKBP genes, including *CsaV3_7G026580*, *CsaV3_4G037510*, *CsaV3_3G007570*, *CsaV3_4G002650*, *CsaV3_1G007080*, *CsaV3_3G036610*, and *CsaV3_6G045680*, were significantly downregulated in both resistant and susceptible cucumber materials, and the *CsaV3_4G002230* gene was only significantly downregulated in the susceptible material (Figure 8f). Under angular leaf spot stress, the *CsaV3_7G026580* gene was significantly upregulated in both resistant and susceptible materials, whereas the *CsaV3_1G007080* gene was significantly downregulated in both resistant and susceptible materials. The *CsaV3_1G015840* gene was only significantly downregulated in the resistant material, and the *CsaV3_3G036610* gene was only significantly downregulated in the susceptible material (Figure 8g). Under root-knot nematode stress, two FKBP genes, *CsaV3_1G007080* and *CsaV3_3G036610*, were significantly upregulated in both resistant and susceptible cucumber materials (Figure 8h).

### 3.10. Regulation Patterns of Cucumber FKBP Genes under Stresses

**Figure 9:**
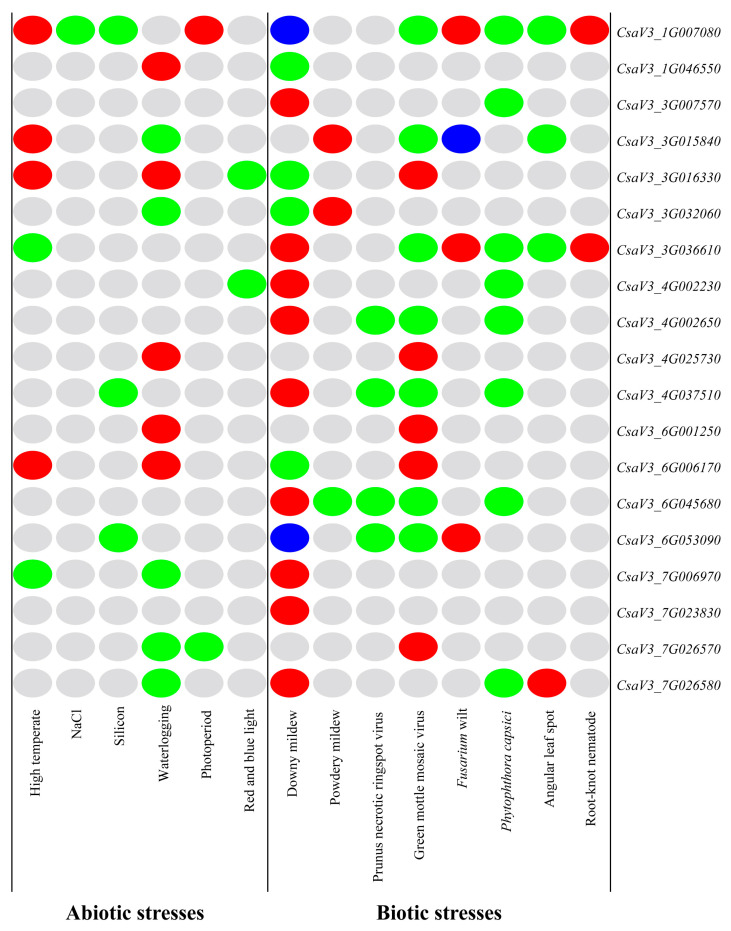
An expression pattern heatmap of the cucumber FKBP genes under abiotic and biotic stresses. The gray color represented unchanged expression, red represented upregulated expression, green represented downregulated expression, and blue represented both upregulated and downregulated expression.

Based on the above expression profiling analysis of the cucumber FKBP family genes, the differentially expressed FKBP genes were classified and labeled, and the relevant heatmap was drawn (Figure 9). It showed that 19 cucumber FKBP genes were all involved in the stress responses; among which, the *CsaV3_1G007080* gene was differentially expressed under the largest number of stresses, including 10 types of stresses, indicating that the *CsaV3_1G007080* gene was actively involved in the stress response. The differentially expressed gene in response to the lowest number of stresses was *CsaV3_7G023830*, which was only differentially expressed in response to downy mildew. Some cucumber FKBP genes, such as *CsaV3_6G045680*, *CsaV3_3G007570*, *CsaV3_4G002650*, and *CsaV3_7G023830*, were only differentially expressed under biotic stresses. Most of the cucumber FKBP genes were differentially expressed under abiotic and biotic stresses, but the expression patterns were different, which could provide references for further research on the biological functions of cucumber FKBP genes.

### 3.11. Protein–Protein Interaction Analysis of Cucumber FKBP Proteins and the CsaV3_1G007080 Protein

**Figure 10:**
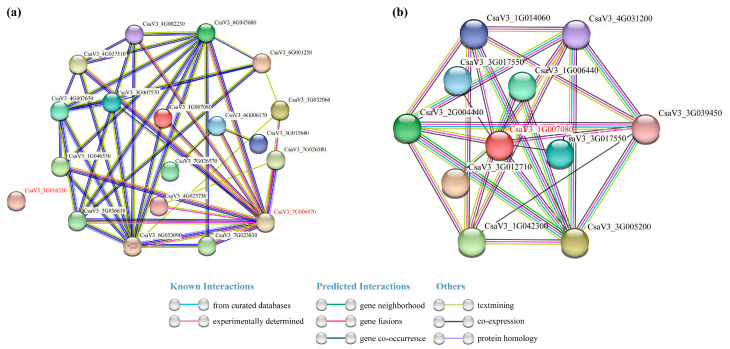
Protein–protein interaction analysis. (**a**) The interaction network of the cucumber FKBP proteins. (**b**) The interaction network between CsaV3_1G007080 and other cucumber proteins.

In order to further study the cucumber FKBP family genes, the interacting proteins of the cucumber FKBP family proteins and CsaV3_1G007080 protein were predicted by the online website STRING (Figure 10). The prediction results of the interacting proteins in the cucumber FKBP gene family showed that CsaV3_3G061330 did not interact with any of the other 18 FKBP proteins, while CsaV3_7G006970 interacted with 13 FKBP proteins (Figure 10a). Nine cucumber proteins interacted with CsaV3_1G007080, including CsaV3_1G006440, CsaV3_1G006440, CsaV3_1G042300, CsaV3_2G004440, CsaV3_3G012710, CsaV3_3G017550, CsaV3_3G005200, CsaV3_3G039540, and CsaV3_4G031200 (Figure 10b).

## 4. Discussion

Plants encounter a variety of stresses during growth, which will decrease the quality and yield of plants and directly lead to plant death in severe cases [61,62]. FKBP is a relatively conserved gene family comprised of proteins with PPlase activity, which plays an important role in response to stress during plant growth and development [25]. In recent years, with the rapid development of next-generation sequencing technology, a lot of plant genomes have been gradually published, and more and more bioinformatics resources are currently available [63]. The FKBP gene family has been identified in many plant species, such as *Arabidopsis* [6], rice [16], maize [17], tomato [18], wheat [19], strawberry [20], peach [21], apple [22], and so on. Although cucumbers are an important vegetable that is widely grown around the world and was the first vegetable crop to finish its entire genome sequencing [33], the genome-wide identification of the FKBP gene family in cucumber has not been conducted, which greatly limits the research on the biological function of FKBP genes in cucumber. Therefore, here, the identification and expression profiling of the cucumber FKBP gene family were performed, which will provide reference for further research on the biological functions of cucumber FKBP genes and provide favorable genes for cucumber resistance breeding.

In this study, the FKBP gene family was identified for the first time in cucumber based on the latest cucumber genome information. A total of 19 FKBP genes were identified in cucumber, which was less than the number of FKBP family genes in *Arabidopsis* (22) [6], rice (29) [16], maize (30) [17], tomato (24) [18], wheat (71) [19], strawberry (23) [20], peach (21) [21], and apple (38) [22]. The number of FKBP genes in different plants was diverse, which may be related to the evolution of plants [64]. The phylogenetic tree analysis of the 19 FKBP genes divided them into three subgroups, namely GROUP I, GROUP II, and GROUP III, which was same with the results of the phylogenetic analysis of the FKBP family genes in maize [17], tomato [18], wheat [19], strawberry [20], and apple [22]. The phylogenetic analysis of FKBP proteins among cucumber, *Arabidopsis*, and rice showed that the more orthologous genes were found between cucumber and *Arabidopsis* but not between cucumber and rice. This may be because cucumber and *Arabidopsis* are both dicotyledon plants and have a closer genetic relationship. The gene duplication analysis of the cucumber FKBP gene family showed that there was one pair of segmental duplication and one pair of tandem duplication, indicating that the expansion of cucumber FKBP genes mainly results from segmental and tandem duplications. This phenomenon is also common in other plant gene families [65,66]. The synteny analysis of the FKBP family genes in cucumber, *Arabidopsis*, and rice found that 12 kinds of syntenic relationships were detected between cucumber and *Arabidopsis* FKBP genes, and 3 kinds of syntenic relationships were observed between cucumber and rice FKBP genes, indicating that these genes may have partially similar functions [67].

High-throughput sequencing technology has become increasingly advanced, and the cost of transcriptome sequencing has decreased [68]. Researchers have performed a large-scale transcriptome sequencing of cucumber and finally formed cucumber transcriptome sequencing big data. The transcriptome sequencing data have been verified by qRT-PCR analysis and peer review, which have been widely recognized [69,70]. Therefore, making full use of these transcriptome data is conducive to improving research efficiency and reducing costs. In this study, the expression patterns of 19 cucumber FKBP genes in different tissues and under different stresses were analyzed based on the published cucumber transcriptome sequencing big data.

In previous studies, it has been reported that the FKBP genes play important roles in the process of plant growth and development. For example, FKBP12 interacted with the CONSTANS protein to affect flowering in *Arabidopsis* [71]. The *Arabidopsis FKBP42* gene promoted stamen elongation, anther dehiscence, and pollen maturation (to a lesser extent) and was required for seed development [72]. The *Arabidopsis FKBP15-1/15–2* genes were expressed prominently in the vascular bundles of the root basal meristem region [73]. In this study, the expression analysis of the 19 cucumber FKBP family genes in 10 types of tissues was conducted, which revealed that the 19 cucumber FKBP genes were expressed in different tissues. Some FKBP genes were expressed in all tissues, and some FKBP genes were specifically expressed in some tissues, indicating that these FKBP genes exhibited tissue-specific expression patterns. The tissue-specific expression patterns of these FKBP family genes in different tissues cooperatively regulate the plant growth and development of cucumber.

In plants, the FKBP genes also play important roles during abiotic and biotic stress responses, including heat, cold, drought, salt, and pathogen infection stresses. For example, wheat TaBI-1.1 regulated the heat tolerance by interacting with TaFKBP62 [74]. In *Arabidopsis*, NBR1 mediated its degradation during heat stress by interacting with ROF1 [75]. ROF1 interacted with phosphatidylinositol-3-phosphate [PI(3)P] and phosphatidylinositol-3,5-bisphosphate [PI(3,5)P_2_] through its FKBD domains under osmotic/salt stress [76]. Overexpressing *Polytrichastrum alpinum PaFKBP12* in *Arabidopsis* showed enhanced resistances to salt, heat, and drought treatments [77]. In *Arabidopsis*, *AtFKBP15-1* positively increased the plant resistance to Phytophthora infection [78]. The *AtFKBP65* gene induced callose accumulation in the cell wall under *Pseudomonas syringe* infection [23]. In our study, the expression profiling analysis of the cucumber FKBP family genes under abiotic stresses showed that more differentially expressed FKBP genes were identified under high-temperature and waterlogging stresses. It was worth noting that the *CsaV3_1G007080* and *CsaV3_3G015840* genes were increased after high-temperature treatment for 3 h and then declined; this phenomenon was also found in maize FKBP genes, such as *ZmFKBP15-3* (*GRMZM2G031204_P01*) and *ZmFKBP16-4* (*GRMZM2G001956_P01*) [17]. The lesser FKBP genes were differentially expressed genes under salt, the photoperiod, and different ratios of blue and red light, with only three, two, and two differentially expressed FKBP genes, respectively, indicating that the cucumber FKBP family genes did not actively respond to these stresses. In addition to abiotic stress, we also analyzed the expression patterns of the cucumber FKBP genes under biotic stresses. More FKBP genes were differentially expressed in response to downy mildew, the green mottle mosaic virus, and *Phytophthora capsica*, which indicated that these cucumber FKBP genes actively responded to these stresses and played a certain role in resisting pathogenic microorganisms. The functional characteristics analysis of the cucumber FKBP genes revealed that all 19 cucumber FKBP genes were differentially expressed under abiotic and biotic stresses. Among them, the *CsaV3_1G007080* gene was differentially expressed under the largest number of stresses (10 types of abiotic and biotic stresses), including high temperature, salt, silicon, photoperiod, downy mildew, green mottle mosaic virus, *fusarium* wilt, *Phytophthora capsica*, angular leaf spot, and root-knot nematode. However, the expression patterns of the *CsaV3_1G007080* gene in response to different stresses were different, including upregulation, downregulation, and both upregulation and downregulation phenomena, which indicated that the *CsaV3_1G007080* gene has various roles in cucumber resistance to stresses. The specific biological function of the *CsaV3_1G007080* gene could be further verified by gene knockout or overexpression.

## 5. Conclusions

In this study, 19 FKBP family genes were systematically identified and characterized in cucumber, which were distributed on chromosomes 1, 3, 4, 6, and 7 and divided into three subgroups. The members of each subgroup were basically conserved, and the gene structure and conserved motifs differed among the different subgroups. The synteny analysis revealed that 12 kinds of syntenic relationships were detected between cucumber and *Arabidopsis* FKBP genes, and 3 kinds of syntenic relationships were observed between cucumber and rice FKBP genes. The tissue-specific expression analysis showed that the cucumber FKBP family genes were specifically expressed in different tissues, which synergistically regulated the cucumber growth and development. The expression profile analysis of the cucumber FKBP genes under 13 types of stresses showed that the *CsaV3_1G007080* gene was differentially expressed under abiotic stresses (high temperature, NaCl, silicon, and photoperiod) and biotic stresses (downy mildew, green mottle mosaic virus, *Fusarium* wilt, *phytophthora capsica*, angular leaf spot, and root-knot nematode), which indicated that the *CsaV3_1G007080* gene played an important role in the growth and development of cucumber. In this study, the expression patterns of the cucumber FKBP genes were analyzed with cucumber transcriptome sequencing big data, which could effectively identify the favorable FKBP genes. These findings might be useful for further functional research on cucumber FKBP genes and will aid in the further breeding of resistant varieties of cucumber.

## Figures and Tables

**Table 1:** The physiochemical characteristics of 19 cucumber FKBP family genes.

Gene ID	CDS Size (bp)	Number of Amino Acid (aa)	Molecular Weight (kDa)	Theoretical pI	Instability Index	Aliphatic Index	Grand Average of Hydropathicity	Prediction of Subcellular Location
*CsaV3_1G007080*	759	252	26.96	6.76	48.72	81.98	−0.25	Chloroplast
*CsaV3_1G046550*	678	225	24.48	7.89	53.13	81.56	−0.11	Chloroplast
*CsaV3_3G007570*	783	260	29.38	9.17	60.58	68.19	−0.517	Chloroplast
*CsaV3_3G015840*	1728	575	64.27	5.23	35.08	76.63	−0.69	Peroxisome
*CsaV3_3G016330*	1524	507	56.14	5.55	43.65	61.28	−1.044	Nucleus
*CsaV3_3G032060*	1110	369	42.17	5.61	36.60	73.25	−0.66	Cytoskeleton
*CsaV3_3G036610*	696	231	24.71	9.41	52.50	89.44	−0.142	Chloroplast
*CsaV3_4G002230*	774	257	27.95	8.90	35.49	76.61	−0.248	Chloroplast
*CsaV3_4G002650*	684	227	24.34	5.97	50.79	91.06	−0.116	Chloroplast
*CsaV3_4G025730*	1866	621	69.96	5.32	43.73	71.26	−0.567	Nucleus
*CsaV3_4G037510*	684	227	24.75	9.49	60.44	78.59	−0.483	Nucleus
*CsaV3_6G001250*	564	187	20.04	6.77	31.63	78.34	−0.506	Nucleus
*CsaV3_6G006170*	1692	563	62.13	5.42	48.46	61.28	−0.956	Nucleus
*CsaV3_6G045680*	648	215	22.57	8.63	43.99	85.26	0.021	Chloroplast
*CsaV3_6G053090*	645	214	22.40	9.23	31.06	88.88	−0.01	Chloroplast
*CsaV3_7G006970*	339	112	11.94	7.76	30.67	73.93	−0.171	Chloroplast
*CsaV3_7G023830*	576	191	21.73	5.76	43.00	93.77	−0.192	Cytoplasm
*CsaV3_7G026570*	1626	541	60.17	5.45	25.01	81.48	−0.554	Cytoplasm
*CsaV3_7G026580*	501	166	17.86	6.30	36.12	78.67	−0.375	Cytoplasm

**Table 2:** The information of ten motifs in cucumber FKBP proteins.

Motif	Sequence	Number of Amino Acids	Pfam Annotation
motif 1	GVKGMKVGEKRRLTIPPELGYG	22	FKBP
motif 2	DDGRPFKFRLGEGQVIKGWDE	21	FKBP
motif 3	PNIPPNATLVFDVELVSV	18	FKBP
motif 4	PKDGDEVKVHYTGKLEDGTVF	21	FKBP
motif 5	QAKALKNPCNLNNAACKLKLKEYKEAEKLCTKV LELDSSNVKALYRRGQAYIQLGDLDLAEEDIKKA	67	-
motif 6	IPPSEYTTTPSGLKYYDIKVGSGP	24	-
motif 7	IEAAGKKKEEGNVLFKEGKFERASKRYEKAVRYIEYDSSF	40	-
motif 8	MGFWGIEVKPGKPFTQKFDDFKGKLRISQATLGFGSAKEKSILQCN	46	-
motif 9	VGNKSPIFLCSLFPEKIECCPLDLEFEEDEEIIFSVIGPRSIHLSGYFLGNCRH	54	-
motif 10	GVLKKILKEGEGWE	14	-

## Data Availability

Not applicable.
